# The emerging role of heparanase in cardiovascular diseases: Pathophysiology, clinical outcomes, and therapeutic perspectives

**DOI:** 10.1007/s11239-025-03208-w

**Published:** 2026-01-28

**Authors:** Emrah Bayam, Macit Kalçık, Ahmet Seyfeddin Gürbüz, Mehmet Özkan

**Affiliations:** 1 Department of Cardiology, University of Medical Sciences, Kartal Kosuyolu High Specialty Training and Research Hospital, İstanbul, Turkey; 2 Department of Cardiology, Faculty of Medicine, Hitit University, Çorum, Turkey; 3 Department of Cardiology, Necmettin Erbakan University Meram Medicine Faculty, Konya, Turkey; 4 Division of Health Sciences, Ardahan University, Ardahan, Turkey

**Keywords:** Heparanase, Atherosclerosis, Thrombosis, Valvular heart disease, Pulmonary hypertension

## Abstract

Endothelial dysfunction, inflammation, atherosclerosis, thrombosis, and extracellular matrix remodeling represent the key pathophysiological processes underlying cardiovascular diseases. Heparanase, the sole mammalian endo-β-D-glucuronidase that cleaves heparan sulfate chains, serves as a key mediator linking these interrelated processes. Through glycocalyx disruption, it enhances vascular permeability, facilitates inflammatory cell recruitment, and promotes tissue factor–driven thrombosis, thereby promoting vascular inflammation and a prothrombotic state. The enzyme contributes to multiple cardiovascular pathologies, including atherosclerosis, thrombosis, restenosis, calcific valvular heart disease, diabetic cardiomyopathy, pulmonary vascular remodeling, and ischemia–reperfusion injury. Notably, its effects are context-dependent, reflecting a “dual role”: excessive activity enhances vascular injury and plaque instability, whereas basal expression may support cardiomyocyte survival and adaptive remodeling under stress conditions. This review discusses molecular mechanisms and therapeutic implications, integrating data from experimental and clinical studies to provide a unified understanding of heparanase biology in the cardiovascular system. Emphasis is placed on translational prospects, highlighting heparanase modulation as both a diagnostic biomarker and a potential therapeutic target for preventing or attenuating cardiovascular disease progression.

**Graphical Abstract:**

Heparanase acts as a central mediator linking endothelial dysfunction, inflammation, thrombosis, and extracellular matrix remodeling in cardiovascular pathology. By degrading heparan sulfate chains within the vascular glycocalyx and matrix, it increases permeability, promotes inflammatory cell recruitment, and enhances tissue factor–driven coagulation. These mechanisms contribute to the progression of atherosclerosis, thrombosis and restenosis, valvular calcification, diabetic cardiomyopathy, and myocardial injury. The enzyme thus represents a unifying molecular link among diverse cardiovascular disorders, serving both as a pathogenic effector and a potential biomarker and therapeutic target.

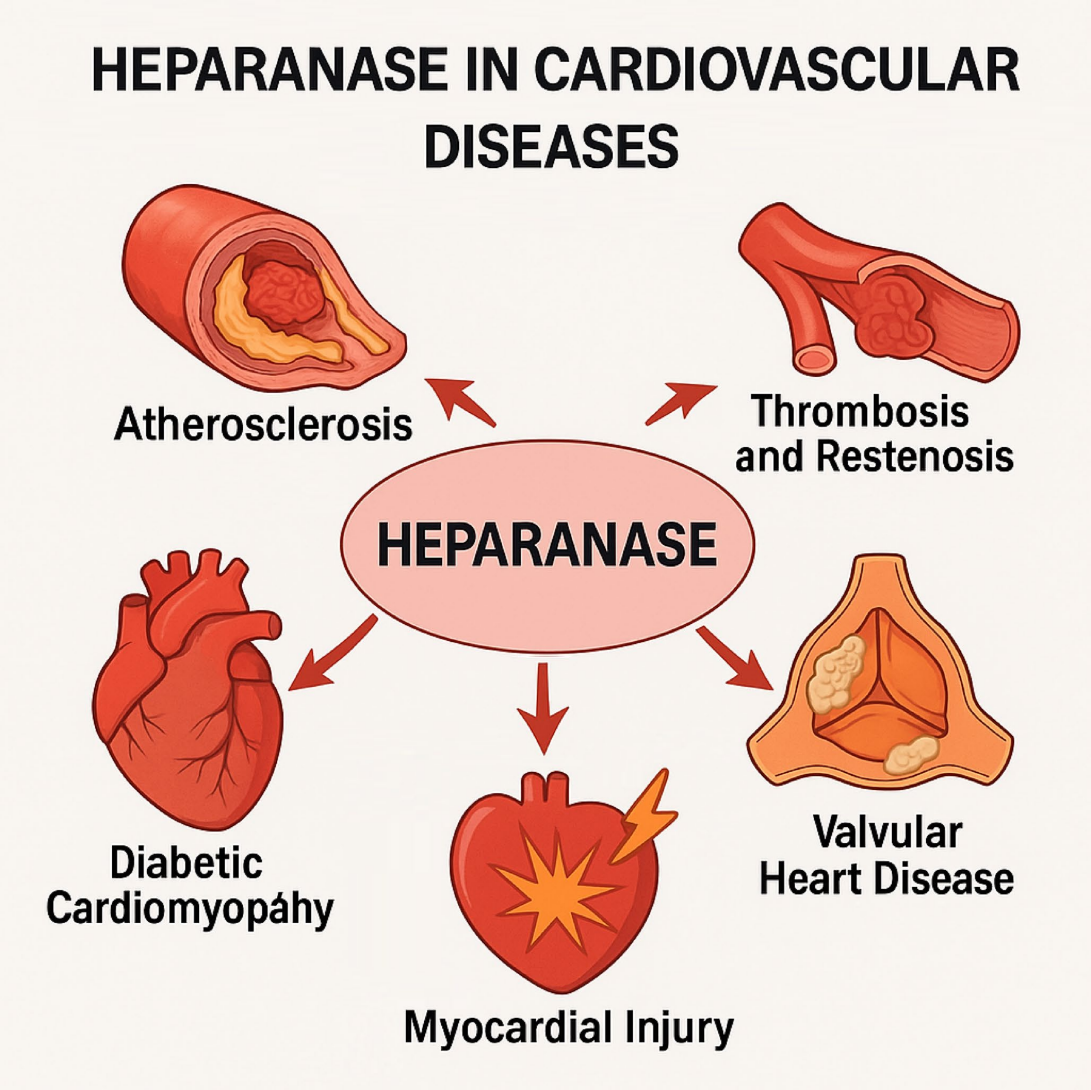

## Introduction

Cardiovascular diseases are the primary cause of death worldwide, accounting for an estimated 18 million deaths annually [1]. Clinical entities such as atherosclerosis, myocardial infarction, stroke, heart failure, and pulmonary hypertension constitute the most common manifestations of this disease group. Endothelial dysfunction, chronic inflammation, extracellular matrix remodeling, and thrombosis are recognized as key mechanisms in their pathophysiology [2].

Heparan sulfate proteoglycans are critical components of the vascular wall, regulating structural integrity, lipoprotein retention, and the storage and release of cytokines and growth factors. Heparanase is the only known mammalian endoglycosidase capable of degrading heparan sulfate chains, thereby playing a central role in vascular biology [3]. Initially investigated in the context of cancer biology due to its role in tumor invasion and metastasis [4], heparanase has more recently been implicated in cardiovascular pathology.

While the biological significance of heparanase was first established in cancer, its effects extend beyond tumor biology. Vascular tissues are particularly susceptible to its activity because the endothelium and subendothelial matrix are rich in heparan sulfate proteoglycans and continuously exposed to shear stress, inflammatory mediators, and coagulation factors. This makes the cardiovascular system a major site where heparanase-driven glycocalyx degradation, inflammation, and thrombosis converge [5, 6].

Within the cardiovascular system, the role of heparanase is complex and multifaceted. On one hand, heparanase degrades heparan sulfate, thereby facilitating leukocyte transmigration, enhancing lipoprotein penetration into the subendothelial space, and promoting atherosclerotic plaque formation [7, 8]. On the other hand, heparanase has been shown to influence intracellular signaling pathways in cardiomyocytes, promoting hypertrophy, autophagy, and survival under stress conditions [9, 10]. Mechanistically, these effects are mediated through activation of intracellular signaling cascades such as the p38 MAPK and PI3K–Akt pathways, which enhance autophagic flux, regulate hypertrophic gene expression, and promote cardiomyocyte survival under ischemic or metabolic stress [11]. This dual role suggests that heparanase may function as both a detrimental and, under specific circumstances, a protective factor in cardiovascular disease progression.

**Fig. 1:**
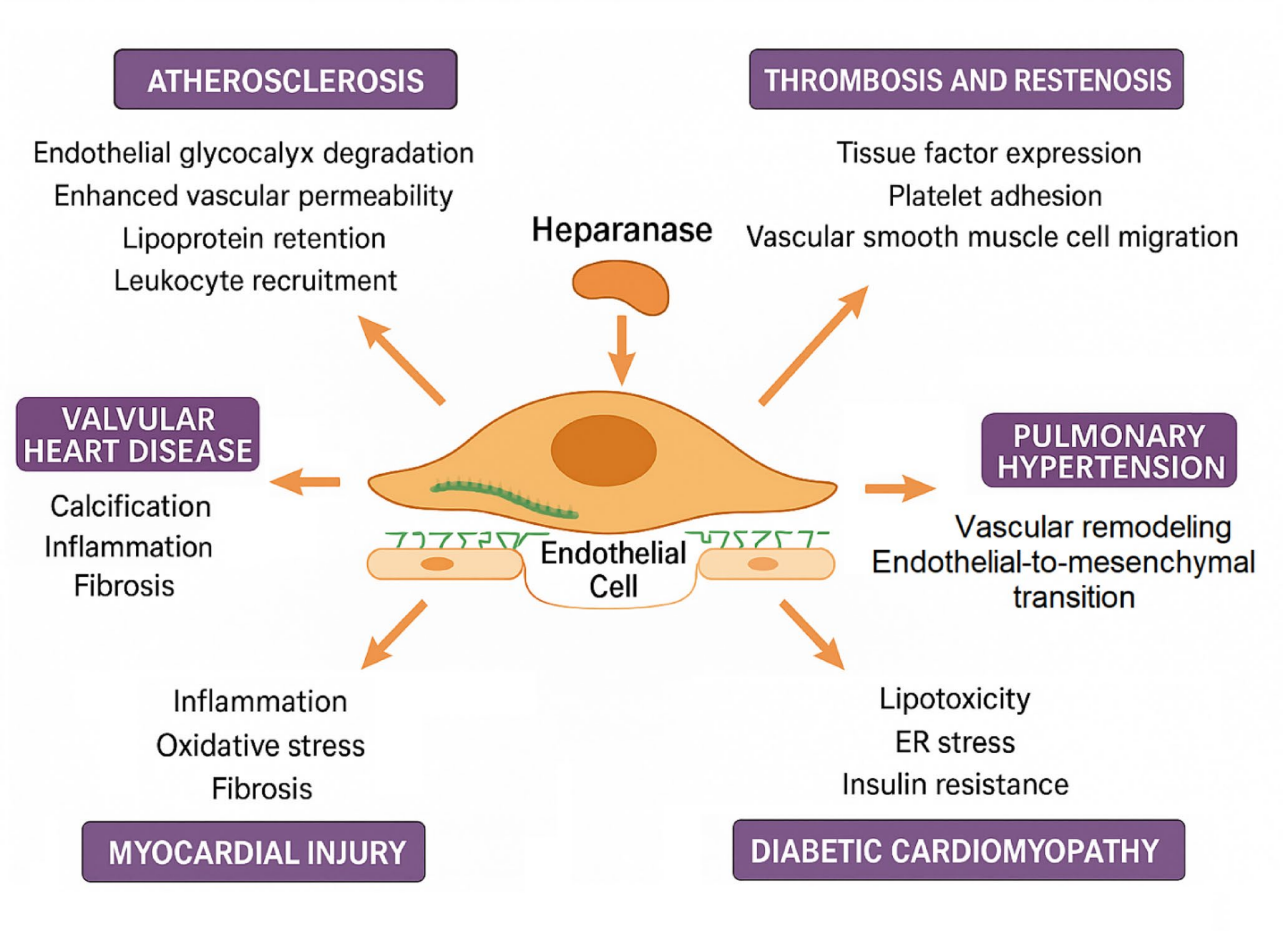
Schematic representation of the multifaceted roles of heparanase in cardiovascular diseases. Heparanase, secreted by endothelial and inflammatory cells, degrades endothelial glycocalyx and extracellular matrix heparan sulfate, triggering downstream vascular and inflammatory responses. In atherosclerosis, it promotes endothelial glycocalyx degradation, lipoprotein retention, and leukocyte recruitment. In thrombosis and restenosis, heparanase enhances tissue factor expression, platelet adhesion, and smooth muscle cell migration. In valvular heart disease, its activity contributes to calcification, inflammation, and fibrosis. In myocardial injury, it amplifies oxidative stress and fibrotic remodeling. In diabetic cardiomyopathy, heparanase drives lipotoxicity, endoplasmic reticulum stress, and insulin resistance. In pulmonary hypertension, it induces vascular remodeling and endothelial-to-mesenchymal transition. Collectively, heparanase links inflammation, endothelial dysfunction, and extracellular matrix remodeling, serving as a central mediator across diverse cardiovascular pathologies

The aim of this review is to provide a comprehensive overview of the current literature regarding the role of heparanase in cardiovascular diseases, with emphasis on its involvement in different pathological processes (Fig. 1) and its potential as a therapeutic target.

## Heparanase in atherosclerosis

Atherosclerosis is a chronic inflammatory disease of the arterial wall characterized by lipid deposition, endothelial dysfunction, immune cell infiltration, and extracellular matrix (ECM) remodeling. It constitutes the fundamental pathological substrate of coronary artery disease, ischemic stroke, and peripheral arterial disease, thereby representing a major cause of global cardiovascular morbidity and mortality [12].

Heparanase, as the sole mammalian endo-β-D-glucuronidase, contributes significantly to the pathogenesis of atherosclerosis by degrading heparan sulfate proteoglycans. This enzymatic activity leads to disruption of the endothelial glycocalyx, enhanced vascular permeability, and increased subendothelial retention of atherogenic lipoproteins [8, 13]. Additionally, the release of heparan sulfate–bound cytokines and growth factors augments vascular inflammation, promoting leukocyte adhesion and transmigration [7, 14].

**Fig. 2:**
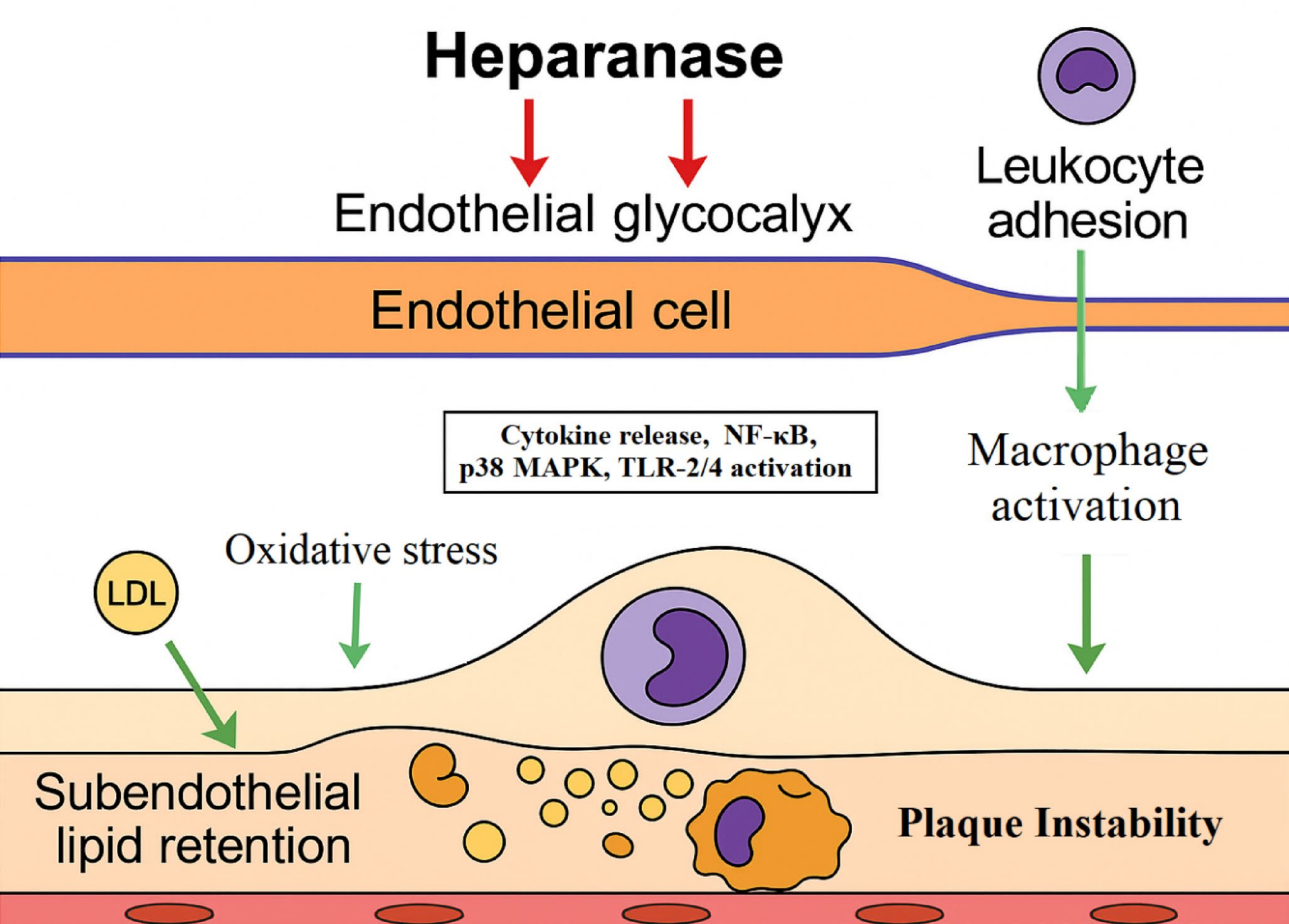
Mechanistic illustration of heparanase-mediated endothelial dysfunction and plaque instability in atherosclerosis. Heparanase-induced degradation of the endothelial glycocalyx increases vascular permeability and facilitates lipid penetration into the subendothelial space, leading to lipid retention and oxidative stress. Endothelial injury promotes cytokine release and activation of NF-κB, p38 MAPK, and TLR-2/4 pathways, enhancing leukocyte adhesion and macrophage activation. These processes amplify inflammation and extracellular matrix degradation, ultimately contributing to plaque progression and instability (LDL: Low-Density Lipoprotein)

Experimental data strongly support the involvement of heparanase in plaque development. Blich et al. demonstrated that macrophage-derived heparanase is indispensable for plaque progression in apolipoprotein E-deficient mice, where its inhibition attenuated lipid accumulation and macrophage infiltration [8]. Other studies have shown that elevated heparanase expression contributes not only to plaque growth but also to destabilization, by weakening the fibrous cap and increasing the risk of rupture [15, 16]. Heparanase expression has been localized to macrophage-rich regions of advanced human plaques, reinforcing its role in promoting plaque vulnerability [17]. Collectively, these findings indicate that macrophage-derived and locally expressed heparanase actively drives plaque progression and destabilization through enhanced inflammation and matrix degradation, reinforcing its mechanistic role in atherosclerosis [15–17]. The proposed molecular and cellular mechanisms underlying heparanase-induced endothelial injury and plaque instability are illustrated in Fig. 2.

Interestingly, clinical findings indicate that the relationship between circulating heparanase levels and atherosclerosis may be more nuanced. In a study conducted by Gurbuz et al., serum heparanase levels were found to be significantly lower in patients with stable coronary artery disease compared to individuals with normal coronary arteries [18]. Moreover, while heparanase levels correlated positively with fasting blood glucose and were higher in diabetic subjects, the overall levels remained lower in stable coronary artery disease patients irrespective of diabetic status. These results suggest that systemic heparanase levels may vary according to plaque stability, being lower in stable disease but elevated in acute and unstable conditions, where vascular injury and inflammation are predominant drivers. This apparent paradox may reflect differences between local and systemic heparanase activity. While vascular tissues in stable plaques may retain high local expression contributing to extracellular matrix remodeling, reduced endothelial turnover and limited glycocalyx shedding could result in lower circulating enzyme levels. Chronic adaptation of the vascular wall and decreased acute inflammatory signaling may further suppress systemic release, despite persistent localized activity within the lesion [19].

These findings imply a dual role of heparanase in atherosclerosis. At the vascular wall level, increased local activity promotes lipoprotein retention, inflammation, and plaque instability [8, 15–17, 20]. However, circulating serum heparanase may decline in stable disease, possibly reflecting a reduced release from vascular compartments or altered regulation in chronic conditions [18]. This dichotomy highlights the complexity of heparanase biology and suggests that both tissue-specific activity and systemic levels must be considered when assessing its role as a biomarker or therapeutic target in atherosclerosis.

In summary, heparanase exhibits a dual role in atherosclerosis that depends on spatial and temporal context. Elevated local activity within the vascular wall contributes to endothelial dysfunction, lipoprotein retention, inflammation, and plaque destabilization. Conversely, lower systemic levels in stable disease may indicate reduced endothelial turnover and a state of chronic vascular adaptation. Recognizing this balance between pathogenic and compensatory functions is essential for interpreting heparanase as both a mechanistic mediator and a potential biomarker of atherosclerotic disease activity. The proinflammatory and prothrombotic consequences of plaque destabilization discussed here are further elaborated in the following section on thrombosis and restenosis, where heparanase-induced glycocalyx degradation and tissue factor activation play central roles. The key molecular and cellular mechanisms linking heparanase activity to atherosclerosis, thrombosis, and restenosis are summarized in Table 1.

**Table 1:** Mechanistic and functional roles of heparanase in Atherosclerosis, thrombosis and restenosis

Mechanism/Pathway	Key Findings	Functional Consequences	References
NF-κB, p38 MAPK, TLR-2/4 activation	Macrophage activation and cytokine release	Promotes plaque inflammation and progression	[8, 15, 19]
Glycocalyx degradation	Lipoprotein retention and endothelial dysfunction	Increased vascular permeability and atherogenesis	[6, 21]
Macrophage-derived heparanase	ECM breakdown in plaques	Plaque instability and rupture risk	[28]
TF–TFPI axis, p38 MAPK, NF-κB	TF upregulation, TFPI release	Procoagulant shift and thrombus formation	[23, 24]
Platelet activation + VEGF/TGF-β release	Chemokine mobilization, SMC proliferation	Neointimal hyperplasia and restenosis	[27]

**Abbreviations**: NF-κB: Nuclear factor kappa-light-chain-enhancer of activated B cells p38 MAPK: p38 mitogen-activated protein kinase TLR: Toll-like receptor ECM: Extracellular matrix TF: Tissue factor TFPI: Tissue factor pathway inhibitor VEGF: Vascular endothelial growth factor TGF-β: Transforming growth factor beta SMC: Smooth muscle cell

## Heparanase in thrombosis and restenosis

Thrombosis is one of the most life-threatening sequelae of atherosclerotic plaque rupture, leading to acute coronary syndromes (ACS) and ischemic complications. Restenosis, conversely, represents a frequent adverse event following percutaneous coronary intervention (PCI), mainly driven by vascular injury, inflammation, and smooth muscle cell proliferation [18]. Accumulating evidence implicates heparanase as a central mediator in both thrombosis and restenosis.

Mechanistically, heparanase exerts prothrombotic effects by degrading the endothelial glycocalyx, thereby exposing adhesion molecules and tissue factor that accelerate platelet adhesion and coagulation cascade activation [21]. Furthermore, it promotes the release of tissue factor pathway inhibitor (TFPI), reducing endogenous anticoagulant control and tipping the hemostatic balance toward thrombosis [22].

Mechanistically, heparanase interacts directly with the tissue factor (TF)–TFPI axis. The enzyme enhances TF expression via activation of the p38 MAPK and NF-κB signaling pathways, thereby amplifying the procoagulant potential of endothelial and vascular smooth muscle cells. Concurrently, heparanase promotes the dissociation and inactivation of TFPI from the endothelial surface, diminishing its inhibitory effect on the TF–factor VIIa complex. This dual modulation results in accelerated thrombin generation and fibrin formation, defining heparanase as a key regulator of coagulation under inflammatory and atherothrombotic conditions [23, 24].

Experimental studies demonstrated that plasma heparanase activity correlates with enhanced thrombin generation and reduced clotting time [25], while also mobilizing chemokines such as monocyte chemoattractant protein-1 (MCP-1) and growth factors including vascular endothelial growth factor (VEGF) and transforming growth factor-β (TGF-β) that recruit leukocytes and vascular smooth muscle cells to the site of injury [26]. This dual effect fosters both thrombus propagation and neointimal hyperplasia, the hallmarks of restenosis [27].

Clinical data reinforce these experimental insights. Osterholm et al. reported increased heparanase expression within human atherosclerotic plaques, where it was associated with plaque destabilization and a prothrombotic microenvironment [28]. More directly, Gurbuz et al. observed that serum heparanase levels are significantly elevated in patients with ST-segment elevation myocardial infarction (STEMI) presenting with high thrombus burden compared to those with lower burden [29]. Importantly, in this prospective study of 187 patients undergoing primary PCI, heparanase levels also predicted the no-reflow phenomenon, further linking the enzyme to adverse thrombotic complications during coronary interventions. However, whether heparanase acts as an independent predictor or simply correlates with thrombus burden remains uncertain. Multivariable analyses did not confirm it as an independent predictor, suggesting that its elevation may primarily reflect the extent of endothelial injury, inflammation, and platelet activation rather than serving as a direct causal determinant. Further studies with larger cohorts and mechanistic endpoints are warranted to clarify its predictive value. These results stand in contrast to their earlier findings in stable coronary artery disease, where serum heparanase was paradoxically lower compared to healthy controls [18]. Collectively, these observations suggest a context-dependent regulation of heparanase, with increased systemic activity in acute thrombotic states (STEMI with high thrombus burden) and suppressed levels in chronic stable disease.

While thrombosis represents the acute consequence of plaque rupture and endothelial injury, restenosis reflects a chronic response to vascular intervention, characterized by smooth muscle proliferation and remodeling. Heparanase contributes to both processes through distinct yet overlapping mechanisms.

Overexpression of the enzyme in vascular smooth muscle cells promotes neointimal thickening and enhances angiogenesis after arterial injury [27]. Conversely, pharmacologic inhibition of heparanase reduces neointimal hyperplasia and attenuates restenotic lesion size in experimental models [20, 30]. In clinical settings, persistent elevation of plasma heparanase after stent implantation has been associated with recurrent restenosis, indicating that systemic heparanase may not only reflect thrombotic activity but also ongoing vascular remodeling [31].

Taken together, the available evidence highlights heparanase as both a pro-thrombotic enzyme and a mediator of restenosis, acting through its capacity to modulate coagulation, inflammation, and vascular remodeling. The contrasting observations between stable CAD and STEMI [18, 29] further emphasize that the enzyme’s role is highly dependent on the underlying pathophysiological state, underscoring its potential as both a biomarker and therapeutic target in cardiovascular disease. Similar procoagulant and inflammatory mechanisms are revisited in the sections on reperfusion injury and valvular heart disease, illustrating the shared pathogenic pathways through which heparanase contributes to cardiovascular complications.

## Heparanase in reperfusion injury

Reperfusion injury represents a setting in which the enzymatic degradation of the extracellular matrix and endothelial glycocalyx by heparanase becomes particularly relevant. This process facilitates vascular leakage, leukocyte adhesion, and inflammatory amplification during the restoration of blood flow.

Reperfusion injury represents a paradoxical phenomenon in which the restoration of blood flow to previously ischemic tissue triggers additional structural and functional damage. In the heart, reperfusion injury is a critical determinant of myocardial infarction outcomes, reperfusion arrhythmias, microvascular dysfunction, and ultimately infarct size [32]. The pathophysiology involves oxidative stress, calcium overload, mitochondrial dysfunction, inflammatory cell infiltration, and ECM degradation [33, 34]. Heparanase has emerged as a key player in this context, acting both as a mediator of tissue damage and, under certain conditions, as a potential adaptive factor.

During ischemia, cellular stress and hypoxia induce the upregulation of heparanase in cardiomyocytes, endothelial cells, and infiltrating immune cells [35]. Upon reperfusion, the enzyme promotes degradation of heparan sulfate within the endothelial glycocalyx and ECM, leading to increased vascular permeability, leukocyte extravasation, and amplified inflammation [36, 37]. This cascade exacerbates myocardial edema and promotes further tissue injury. In addition, heparanase-induced release of stored cytokines and growth factors contributes to a pro-inflammatory milieu that perpetuates myocardial necrosis and remodeling [7].

Experimental studies support the involvement of heparanase in myocardial ischemia–reperfusion injury. In murine models, genetic or pharmacological inhibition of heparanase reduced infarct size, inflammatory cell recruitment, and oxidative stress, while preserving endothelial glycocalyx integrity and microvascular function (35, 38). Conversely, heparanase activity promoted leukocyte adhesion and endothelial dysfunction, exacerbating tissue injury. These findings suggest that modulation of heparanase may represent a therapeutic strategy for limiting ischemia–reperfusion–induced cardiac damage [23, 39].

Heparanase functions in a dose- and context-dependent manner, being deleterious when excessively activated but potentially adaptive at basal levels. Low-level heparanase activity may confer cardioprotective effects by inducing autophagy and enhancing cell survival pathways during hypoxic stress [40]. Experimental data indicate that this switch between deleterious and adaptive effects depends largely on enzyme concentration, timing, and duration of expression. Transient or low-level heparanase activity activates PI3K–Akt and autophagy-related pathways, supporting cardiomyocyte survival and remodeling under stress. In contrast, sustained or high-level expression promotes endothelial glycocalyx degradation, p38 MAPK activation, and inflammatory amplification, leading to vascular injury and microvascular dysfunction [11].

Clinically, elevated plasma heparanase has been detected in patients with acute myocardial infarction, correlating with markers of reperfusion injury such as troponin release and impaired microvascular perfusion [8, 11]. These findings underscore its potential as a biomarker for identifying patients at higher risk of adverse remodeling following reperfusion therapy.

In summary, heparanase plays a multifaceted role in ischemia-reperfusion injury, mediating endothelial dysfunction, inflammatory amplification, and structural remodeling. Its inhibition represents a promising therapeutic strategy to reduce reperfusion-induced myocardial damage, though further studies are needed to clarify its context-dependent protective versus deleterious effects.

## Heparanase in native valvular disease

Valvular heart disease (VHD) is an important cause of morbidity and mortality, with calcific aortic valve stenosis (CAVS) being the most prevalent form in the aging population. The pathogenesis of CAVS shares similarities with atherosclerosis, including endothelial dysfunction, lipid infiltration, chronic inflammation, and extracellular matrix remodeling [41]. Recent evidence suggests that heparanase plays a significant role in these processes, particularly in the progression of aortic valve calcification.

Heparanase is abundantly expressed in calcified aortic valves, predominantly localized in valvular interstitial cells (VICs) and inflammatory infiltrates [42]. Its enzymatic degradation of heparan sulfate facilitates the release of bound growth factors such as VEGF and transforming growth factor-β (TGF-β), both of which contribute to osteogenic differentiation of VICs and subsequent calcification [43]. Moreover, heparanase-induced matrix remodeling enhances infiltration of macrophages and T lymphocytes, amplifying the inflammatory milieu characteristic of stenotic valves [44].

Experimental studies have demonstrated that heparanase expression is upregulated in response to pro-inflammatory cytokines within VICs, promoting osteogenic pathways and mineralization [45]. Clinically, elevated heparanase expression has been correlated with the severity of valvular calcification and reduced valvular area, indicating its potential as both a biomarker of disease progression and a therapeutic target [46].

The study by Rosen et al. provided critical insight into the mechanistic involvement of heparanase in CAVS. Their findings demonstrated that increased heparanase expression in aortic valves is not merely a bystander phenomenon but actively contributes to disease progression through ECM remodeling and promotion of calcification [46]. These results suggest that, similar to its role in atherosclerosis and thrombosis, heparanase functions as a driver of valvular pathology, highlighting the enzyme as a novel target for therapeutic intervention in VHD.

## Heparanase in prosthetic valve thrombosis

Heparanase, beyond its canonical role in remodeling heparan sulfate, has emerged as a modulator of coagulation and thrombosis, plausibly relevant to valvular pathobiology where blood-material interfaces and endothelial injury amplify thrombotic risk. In mechanical prostheses, the combination of foreign surface, altered flow, and patient-related factors creates a milieu in which heparanase’s procoagulant and pro-inflammatory activities could promote thrombus formation and impede antithrombotic therapy responses [47].

In the context of prosthetic valve thrombosis, Bayam et al. demonstrated that patients with thrombotic obstruction of mechanical valves exhibit significantly higher circulating heparanase activity compared with both non-obstructive prosthesis carriers and healthy controls. Elevated enzyme levels were associated with increased tissue factor expression and heightened thrombin generation, linking heparanase to a procoagulant and inflammatory state [47]. These findings suggest that heparanase not only participates in thrombosis by promoting endothelial and platelet activation but may also serve as a potential biomarker for thrombotic risk in prosthetic valve recipients.

In the same study, unfractionated heparin (UFH) therapy was found to increase circulating heparanase levels in patients with prosthetic valve thrombosis. Post-treatment enzyme activity was higher in those with suboptimal thrombus resolution compared with patients who achieved successful lysis. Although the correlations were modest, this pattern suggests that individuals with elevated baseline heparanase may exhibit relative resistance to UFH therapy, requiring longer treatment durations and demonstrating less favorable responses. These findings reinforce the concept that heparanase contributes to anticoagulant resistance and persistent thrombogenic activity in prosthetic valve thrombosis [47].

These findings position heparanase as: a biomarker signaling thrombus presence and complexity in prosthetic valves; a potential predictor of UFH responsiveness; and a candidate participant in the pathogenesis of PVT, where heightened levels align with obstructive morphology, greater thrombus burden, and recent thromboembolism. From a mechanistic standpoint, Bayam et al. discuss heparanase’s ability to potentiate tissue factor-driven coagulation and factor Xa generation, offering a biologically coherent link between elevated circulating heparanase and a prothrombotic PVT phenotype [47].

Collectively, these observations illustrate how heparanase serves as a molecular bridge between inflammation, calcification, and thrombosis in valvular pathology. By degrading the extracellular matrix and endothelial glycocalyx, the enzyme enhances vascular permeability and leukocyte infiltration, fostering chronic inflammation [48]. This proinflammatory environment promotes osteogenic transformation of valvular interstitial cells through TGF-β and BMP signaling, driving calcific remodeling in native valves. Simultaneously, exposure of tissue factor and release of TFPI from the endothelium amplify thrombin generation and fibrin formation, establishing a direct mechanistic link between inflammatory injury, calcification, and thrombosis.

## Heparanase in diabetic cardiomyopathy

Diabetic cardiomyopathy (DCM) is a distinct clinical entity characterized by structural and functional alterations of the myocardium in patients with diabetes mellitus, independent of coronary artery disease or hypertension. Hallmarks include left ventricular hypertrophy, diastolic dysfunction, interstitial fibrosis, and, at later stages, systolic impairment [49]. Although hyperglycemia, oxidative stress, and advanced glycation end products (AGEs) are central to its pathogenesis, accumulating evidence points to the role of ECM remodeling and inflammatory signaling. Within this context, heparanase has emerged as a key mediator.

Hyperglycemia upregulates heparanase expression in endothelial cells and cardiomyocytes, promoting the cleavage of heparan sulfate proteoglycans in the glycocalyx and ECM [50]. Heparanase promotes the release of fibroblast growth factor (FGF) and transforming growth factor-β (TGF-β) from the extracellular matrix, enhancing profibrotic and remodeling pathways in diabetic myocardium [51]. In experimental models of diabetes, cardiac overexpression of heparanase has been associated with increased myocardial hypertrophy, augmented fibrosis, and impaired diastolic function [11].

In diabetic conditions, AGEs upregulate heparanase expression through receptor for advanced glycation end-products (RAGE)-dependent activation of NF-κB and ERK signaling pathways, leading to endothelial glycocalyx degradation and increased vascular permeability [52]. In turn, heparanase enhances AGE/RAGE signaling by releasing heparan sulfate–bound cytokines and growth factors such as TGF-β and VEGF, thereby amplifying oxidative stress, inflammation, and profibrotic remodeling in the myocardium [53]. This bidirectional interaction establishes a self-perpetuating loop linking hyperglycemia to myocardial fibrosis and diastolic dysfunction.

Interestingly, heparanase may also exert context-dependent protective effects. Studies have reported that low-level heparanase expression enhances autophagy and promotes cardiomyocyte survival under hyperglycemic stress [40]. Moreover, controlled heparanase activity has been linked to improved angiogenesis and adaptive remodeling, suggesting that the enzyme may serve as a “double-edged sword” in DCM pathophysiology [54]. At moderate levels, heparanase may exert protective effects by activating PI3K–Akt and autophagy-related pathways, thereby enhancing cellular survival and maintaining metabolic homeostasis under hyperglycemic stress. However, excessive or sustained heparanase expression triggers activation of p38 MAPK and NF-κB signaling, leading to oxidative stress, inflammatory cytokine release, and extracellular matrix degradation [11, 48]. This molecular divergence underlies the enzyme’s “double-edged sword” behavior in diabetic cardiomyopathy.

Emerging evidence suggests that heparanase overexpression in diabetes is at least partially reversible. Improved glycemic control and reduction of advanced glycation end-products (AGEs) can restore endothelial glycocalyx integrity and lower heparanase levels. Moreover, pharmacological interventions such as heparanase inhibitors (e.g., PG545, SST0001) and SGLT2 inhibitors have been shown to suppress heparanase activity, attenuate inflammation, and reduce myocardial fibrosis in experimental diabetic models [48]. These findings indicate that heparanase represents a modifiable component of diabetic cardiac injury rather than a fixed pathological feature.

Clinical data remain limited, but circulating heparanase levels have been found to correlate with poor glycemic control and higher rates of cardiovascular complications in diabetic populations [18]. These findings align with experimental evidence, supporting a role for heparanase in linking hyperglycemia to adverse myocardial remodeling.

In summary, heparanase contributes to the development of diabetic cardiomyopathy by promoting ECM remodeling, fibrosis, and inflammation, while under certain conditions it may also support adaptive cardiomyocyte survival. Most mechanistic data supporting these concepts originate from experimental animal models, in which heparanase overexpression promotes fibrosis, oxidative stress, and diastolic dysfunction through AGE/RAGE and PI3K–Akt pathways. In contrast, human studies have primarily demonstrated elevated circulating or myocardial heparanase levels correlating with impaired diastolic function and myocardial stiffness rather than direct mechanistic causality. Distinguishing these experimental and clinical perspectives underscores the translational potential of targeting heparanase in diabetic heart disease. Further research is required to clarify the balance between detrimental and protective effects and to assess whether therapeutic inhibition of heparanase could mitigate the structural and functional alterations of the diabetic heart. A comparative overview of heparanase-related mechanisms across valvular, metabolic, and pulmonary cardiovascular disorders is provided in Table 2.

**Table 2:** Mechanistic and functional roles of heparanase in valvular heart Disease, diabetic cardiomyopathy and pulmonary hypertension

Disease Context	Mechanism	Major Effects	References
Calcific Aortic Valve Stenosis	Heparanase-induced VEGF and TGF-β release, NF-κB and BMP signaling activation	VIC osteogenic transformation, calcification and fibrosis	[44–46]
Prosthetic Valve Thrombosis	Elevated circulating heparanase levels	Higher thrombus burden and poor UFH response	[47]
Diabetic Cardiomyopathy	AGE/RAGE → NF-κB, ERK activation	ECM degradation → fibrosis and diastolic dysfunction	[51–53]
Pulmonary Hypertension	TGF-β/Smad and Wnt/β-catenin pathways, TGF-β–mediated fibroblast activation	PASMC proliferation, ECM remodeling and RV fibrosis	[56, 57]

**Abbreviations**: VEGF: Vascular endothelial growth factor TGF-β: Transforming growth factor beta NF-κB: Nuclear factor kappa-light-chain-enhancer of activated B cells BMP: Bone morphogenetic protein VIC: Valvular interstitial cell UFH: Unfractionated heparin AGE: Advanced glycation end-products RAGE: Receptor for advanced glycation end-products ERK: Extracellular signal-regulated kinase ECM: Extracellular matrix TGF-β/Smad: Transforming growth factor beta/Mothers against decapentaplegic homolog signaling pathway Wnt/β-catenin: Wingless-related integration site/beta-catenin signaling pathway PASMC: Pulmonary artery smooth muscle cell RV: Right ventricle

## Heparanase in pulmonary hypertension

Pulmonary hypertension (PH) remains a progressive and life-threatening condition characterized by elevated pulmonary vascular resistance and right heart failure, with limited therapeutic options despite advances in treatment. Its pathogenesis involves endothelial dysfunction, smooth muscle cell proliferation, inflammation, and ECM remodeling [55]. Emerging evidence indicates that heparanase plays an important role in these pathological processes.

One of the central features of PH is vascular remodeling, which encompasses proliferation and migration of pulmonary arterial smooth muscle cells (PASMCs), deposition of ECM, and perivascular inflammation. Heparanase, by degrading heparan sulfate proteoglycans, facilitates the release of growth factors such as fibroblast growth factor-2 (FGF-2) and VEGF, which are potent drivers of PASMC proliferation and angiogenesis [3]. This enzymatic activity also enhances leukocyte infiltration, amplifying vascular inflammation and further exacerbating pulmonary vascular remodeling [7].

In addition to its vascular effects, emerging evidence suggests that heparanase may directly influence right ventricular (RV) structure and function. Experimental pulmonary hypertension models have demonstrated increased heparanase expression within the RV myocardium, associated with interstitial fibrosis, hypertrophy, and impaired contractility. These changes are thought to result from TGF-β–mediated fibroblast activation and extracellular matrix remodeling, indicating that heparanase contributes not only to pulmonary vascular remodeling but also to secondary right ventricular maladaptation [56, 57].

Animal studies have provided mechanistic insights. Overexpression of heparanase in transgenic mice has been linked to pulmonary vascular thickening and increased right ventricular systolic pressure under hypoxic conditions [56]. Conversely, pharmacological inhibition of heparanase attenuated hypoxia-induced pulmonary hypertension, suggesting a causal role in disease progression [58].

From a molecular standpoint, heparanase also modulates signaling cascades implicated in PH, such as the TGF-β/Smad and Wnt/β-catenin pathways, both of which are known to contribute to PASMC proliferation and vascular remodeling [59]. In addition, cleavage of heparan sulfate by heparanase destabilizes the endothelial glycocalyx, impairing nitric oxide bioavailability and promoting vasoconstriction [21].

Although clinical data remain limited, elevated circulating heparanase has been reported in patients with idiopathic pulmonary arterial hypertension, correlating with markers of disease severity such as pulmonary vascular resistance and N-terminal pro–B-type natriuretic peptide (NT-proBNP) [57]. These observations suggest that heparanase may serve as a biomarker of disease activity and a potential therapeutic target.

In summary, heparanase contributes to the development and progression of pulmonary hypertension by promoting vascular remodeling, inflammation, and endothelial dysfunction. Its inhibition represents a promising therapeutic strategy to mitigate vascular remodeling and improve hemodynamics in patients with PH. An integrative comparison of heparanase-regulated pathways across major cardiovascular diseases is shown in Table 3.

**Table 3:** Comparative summary of heparanase-regulated mechanisms across cardiovascular diseases

Disease	Major Pathways	Key Cellular Effects	Pathophysiological Outcomes	References
Atherosclerosis	NF-κB, p38 MAPK, TLR-2/4	Macrophage activation, ECM degradation, endothelial dysfunction	Plaque progression and instability	[7, 8, 15–17, 20]
Thrombosis/Restenosis	Tissue Factor–TFPI axis, p38 MAPK, NF-κB	TF expression, platelet activation, smooth muscle proliferation	Thrombus formation, neointimal hyperplasia	[21–24, 27–30]
Valvular Heart Disease (CAVS, PVT)	TGF-β/Smad, BMP, NF-κB	VIC osteogenic differentiation, fibrosis, TF release	Valve calcification and thrombosis	[42–47]
Diabetic Cardiomyopathy	AGE/RAGE, PI3K–Akt, p38 MAPK, NF-κB	Autophagy modulation, oxidative stress, fibroblast activation	Myocardial fibrosis and diastolic dysfunction	[40, 48–54]
Pulmonary Hypertension	TGF-β/Smad, Wnt/β-catenin, NF-κB	Endothelial-to-mesenchymal transition, smooth muscle proliferation, RV fibrosis	Vascular remodeling and RV hypertrophy	[55–59]

**Abbreviations**: AGE: Advanced glycation end-products; BMP: Bone morphogenetic protein; CAVS: Calcific aortic valve stenosis; ECM: Extracellular matrix; NF-κB: Nuclear factor kappa-light-chain-enhancer of activated B cells; PI3K: Phosphoinositide 3-kinase; PVT: Prosthetic valve thrombosis; RV: Right ventricle; TF: Tissue factor; TFPI: Tissue factor pathway inhibitor; TGF-β: Transforming growth factor-beta; TLR: Toll-like receptor

## Therapeutic implications of targeting heparanase in CVDs

The growing body of evidence implicating heparanase in atherosclerosis, thrombosis, valvular heart disease, pulmonary hypertension, and ischemia-reperfusion injury highlights the enzyme as a promising therapeutic target in cardiovascular disease (CVD). Therapeutic strategies have largely focused on inhibiting heparanase enzymatic activity, modulating its downstream signaling pathways, or utilizing heparanase-derived biomarkers for risk stratification [20]. The major pharmacological agents and strategies targeting heparanase in cardiovascular disease are outlined in Table 4.

**Table 4:** Potential therapeutic agents targeting heparanase and their mechanisms in cardiovascular diseases

Drug/Agent	Drug Class or Type	Mechanism of Action on Heparanase Pathway	Expected Cardiovascular Effect	Development/Study Status	References
Unfractionated Heparin (UFH)	Classical anticoagulant	Binds to heparanase and inhibits enzymatic degradation of heparan sulfate; partial suppression of tissue factor activation.	Reduces thrombosis and inflammation but increases bleeding risk.	Widely used; indirect inhibitor.	[47, 60]
Low-Molecular-Weight Heparin (LMWH)	Anticoagulant	Partial heparanase inhibition; limits endothelial injury and glycocalyx loss.	Lowers thrombus formation in ACS and PVT.	Clinical use; partial efficacy.	[29, 60]
Non-Anticoagulant Heparin Derivatives (e.g., Roneparstat, Necuparanib)	Heparanase inhibitor (heparin mimetic)	Block heparanase enzymatic site without anticoagulant effect; suppress ECM degradation and inflammatory cytokine release.	Anti-inflammatory and anti-thrombotic without bleeding risk.	Preclinical/early clinical phase.	[30, 61, 62]
PG545 (Pixatimod)	Synthetic sulfated oligosaccharide	Direct heparanase inhibitor; reduces ECM remodeling, TF activation, and leukocyte infiltration.	Prevents vascular inflammation, restenosis, and atherosclerotic progression.	Preclinical cardiovascular models; oncology phase II trials.	[62, 63]
SGLT2 Inhibitors (e.g., Empagliflozin, Dapagliflozin)	Antidiabetic agent	Indirectly suppresses heparanase through improved glycemic control and reduced AGE/RAGE signaling.	Decreases myocardial fibrosis and inflammation in diabetic cardiomyopathy.	Approved; mechanistic cardiovascular studies ongoing.	[48, 53]
Heparanase siRNA/Antisense Oligonucleotides	Gene-silencing therapy	Inhibits transcription of heparanase mRNA; downregulates enzyme expression and its downstream effects.	Reduces vascular inflammation and ECM remodeling.	Experimental; no clinical data yet.	[65]
Small-Molecule Heparanase Inhibitors	Novel pharmacological class	Occupy heparanase catalytic pocket; block substrate binding.	Attenuate vascular remodeling and coagulation activation.	Preclinical; oncology focus, potential cardiovascular use.	[62, 64]

**Abbreviations**: ACS: Acute Coronary Syndrome; AGE: Advanced Glycation End-products; ECM: Extracellular Matrix; LMWH: Low-Molecular-Weight Heparin; PVT: Prosthetic Valve Thrombosis; RAGE: Receptor for Advanced Glycation End-products; SGLT2: Sodium–Glucose Cotransporter 2; siRNA: small interfering RNA; TF: Tissue Factor; UFH: Unfractionated Heparin

Classical anticoagulants such as unfractionated heparin (UFH) and low-molecular-weight heparins (LMWH) exert partial inhibitory effects on heparanase. Beyond their anticoagulant role, these agents interfere with heparan sulfate degradation and subsequent growth factor release, thereby attenuating vascular inflammation and remodeling [60]. Non-anticoagulant heparin derivatives and synthetic sulfated oligosaccharides have been developed to specifically inhibit heparanase without the bleeding risk associated with heparin, showing promising results in preclinical studies [30].

Several heparanase inhibitors have been evaluated in preclinical and clinical studies, primarily within the field of oncology. By reducing ECM remodeling, leukocyte infiltration, and growth factor mobilization, these agents may attenuate atherosclerotic plaque progression, thrombosis, and restenosis [61]. While several heparanase inhibitors such as PG545 and Roneparstat have shown promising anti-inflammatory and antithrombotic effects in experimental models, translating these agents from oncology to cardiology presents notable challenges [62–64]. Differences in tissue pharmacokinetics, endothelial responses, and hemostatic balance complicate dose optimization for cardiovascular applications. Moreover, systemic inhibition of heparanase may impair physiological tissue repair and angiogenesis, necessitating context-specific modulation rather than complete blockade. Addressing these translational and safety issues will be crucial before heparanase inhibitors can be realistically applied in cardiovascular therapeutics.

In addition to translational barriers, systemic inhibition of heparanase may lead to unintended physiological consequences. Because the enzyme participates in normal extracellular matrix remodeling, angiogenesis, and wound healing, prolonged or non-selective inhibition could impair tissue regeneration and vascular repair. Moreover, heparanase contributes to leukocyte migration and immune surveillance; thus, its suppression might alter host defense or inflammatory resolution. These considerations highlight the importance of developing targeted, tissue-specific approaches or transient inhibition strategies to minimize systemic side effects while preserving therapeutic efficacy [53, 65].

RNA interference and antisense oligonucleotides targeting heparanase expression have demonstrated efficacy in experimental models of inflammation and vascular injury. While still in early stages, these approaches could represent future precision therapies for CVD patients with elevated heparanase activity [66].

In addition to therapeutic targeting, circulating heparanase levels may serve as a biomarker for risk prediction. Clinical studies have shown that heparanase correlates with thrombus burden in acute coronary syndromes, predicts adverse outcomes after reperfusion therapy, and associates with treatment resistance in prosthetic valve thrombosis [18, 29, 32]. These observations suggest that monitoring heparanase could guide both therapeutic decisions and prognosis in CVD patients.

Furthermore, circulating heparanase has emerged as a potential biomarker reflecting disease activity across cardiovascular disorders. As noted in earlier sections, plasma heparanase levels rise markedly in acute or inflammatory settings such as STEMI, prosthetic valve thrombosis, and pulmonary hypertension, whereas more stable disease stages may show normalized or even reduced levels [18, 29, 47]. This dynamic pattern suggests that heparanase functions not as a static biomarker, but as a context-dependent indicator of endothelial stress and remodeling. Recognizing this variability is essential when considering its use in diagnosis or treatment monitoring.

**Fig. 3:**
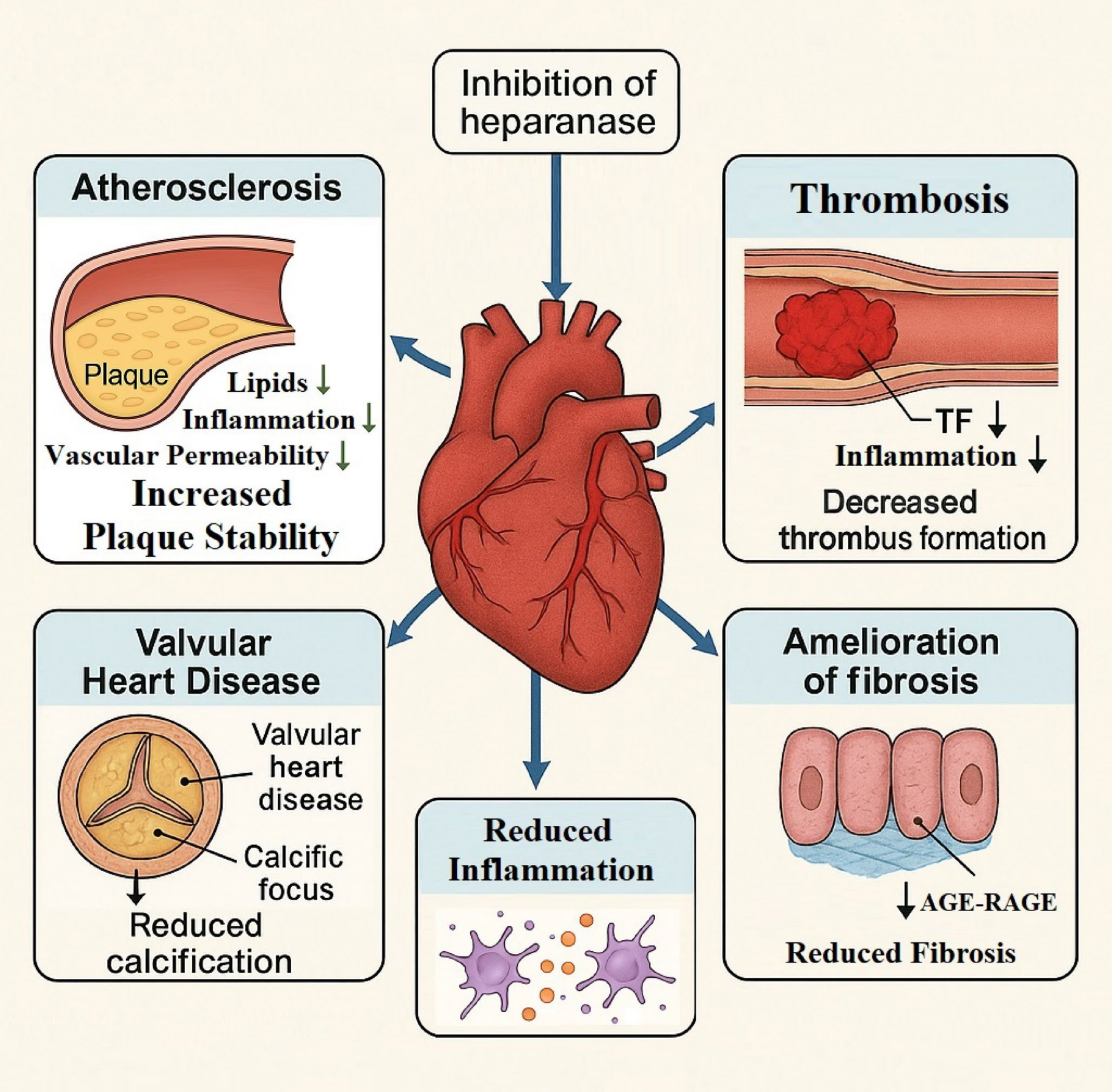
Schematic summary of the cardiovascular benefits associated with inhibition of heparanase. Blocking heparanase activity reduces endothelial injury, inflammation, and extracellular matrix degradation, thereby exerting protective effects across multiple cardiovascular disorders. In atherosclerosis, inhibition decreases lipid accumulation, vascular permeability, and inflammation, leading to enhanced plaque stability. In thrombosis, suppression of tissue factor expression and inflammation results in reduced thrombus formation. In valvular heart disease, heparanase inhibition mitigates inflammatory calcification, lowering valvular stiffness and remodeling. In fibrotic disorders, inhibition attenuates AGE–RAGE–mediated signaling, reducing myocardial fibrosis. Collectively, heparanase inhibition promotes vascular integrity and global cardiovascular protection through anti-inflammatory and antifibrotic mechanisms (TF: Tissue Factor, AGE: Advanced Glycation End-product, RAGE: Receptor for Advanced Glycation End-products)

In summary, emerging therapeutic approaches targeting heparanase show promise in mitigating cardiovascular inflammation and thrombosis. Among them, non-anticoagulant heparin derivatives such as roneparstat and necuparanib appear particularly attractive, as they preserve anti-inflammatory and endothelial-protective actions without increasing bleeding risk. Selective small-molecule inhibitors and agents with indirect heparanase-suppressive effects, including SGLT2 inhibitors, also hold translational potential [11, 53]. These strategies highlight the feasibility of modulating heparanase activity in a disease- and context-specific manner, paving the way for safer cardiovascular applications. The potential therapeutic benefits of heparanase inhibition across major cardiovascular pathologies are summarized in Fig. 3.

## Future perspectives

Heparanase is as a multifaceted enzyme with profound implications for cardiovascular disease. Its ability to degrade heparan sulfate proteoglycans positions it at the crossroads of inflammation, coagulation, vascular remodeling, and fibrosis. Current evidence supports its involvement in a wide spectrum of cardiovascular pathologies, including atherosclerosis, thrombosis, restenosis, valvular heart disease, diabetic cardiomyopathy, pulmonary hypertension, and ischemia-reperfusion injury. Key experimental and clinical findings investigating heparanase in cardiovascular contexts are summarized in Table 5.

**Table 5:** Summary of recent experimental and clinical studies investigating heparanase in cardiovascular diseases (2018–2024)

Study/First Author	Study Type	Population/Model	Main Findings	Cardiovascular Implication	References
Bayam et al. (2018)	Clinical (PVT)	60 patients with prosthetic valve thrombosis	Serum heparanase levels elevated in thrombotic obstruction; higher levels correlated with poor UFH response.	Indicates heparanase as a biomarker of thrombogenicity and UFH resistance.	[47]
Gürbüz et al. (2019)	Clinical (CAD, STEMI)	187 STEMI patients; 82 stable CAD controls	Elevated heparanase in high thrombus burden STEMI; decreased in stable CAD.	Reflects dual regulation—up in acute injury, down in chronic disease.	[18, 29]
Wang et al. (2019)	Experimental (mouse)	Heparanase overexpression and knockout mice	Heparanase protects myocardium from I/R injury at basal levels but aggravates injury when overexpressed.	Supports context-dependent dual role of heparanase in reperfusion.	[10]
Capozzi et al. (2021)	In vitro/translational	Endothelial and platelet models	Heparanase inhibition reduced TF expression and platelet activation.	Confirms therapeutic potential of heparanase inhibitors in thrombosis.	[24]
Wang et al. (2023)	Experimental (PH model)	Hypoxia-induced pulmonary hypertension in rats	Heparanase inhibition decreased RV pressure and vascular remodeling.	Suggests therapeutic value of targeting heparanase in pulmonary hypertension.	[56]
Lee et al. (2024)	Experimental (Diabetes model)	Mouse model of chronic diabetes	Chronic diabetes suppressed physiological heparanase signaling, leading to maladaptive remodeling.	Demonstrates protective basal role lost under diabetic stress.	[11]
Zhang et al. (2024)	Clinical proteomic study	60 patients with chronic thromboembolic PH	Serum heparanase identified as biomarker for disease severity.	Indicates translational diagnostic potential in pulmonary vascular disease.	[57]
Noda et al. (2024)	Experimental (Ischemia/Reperfusion)	Mouse lung I/R model	Heparanase-driven glycocalyx degradation worsened microvascular injury.	Reinforces mechanistic link between glycocalyx loss and reperfusion damage.	[37]

**Abbreviations**: CAD: Coronary Artery Disease; PVT: Prosthetic Valve Thrombosis; UFH: Unfractionated Heparin; STEMI: ST-segment Elevation Myocardial Infarction; I/R: Ischemia–Reperfusion; TF: Tissue Factor; PH: Pulmonary Hypertension; RV: Right Ventricle

From a clinical standpoint, circulating heparanase levels show promise as a biomarker for risk stratification in patients with coronary artery disease, acute coronary syndromes, and prosthetic valve thrombosis. Importantly, the contrasting observations of low heparanase levels in stable coronary artery disease and elevated levels in acute thrombotic states highlight the context-dependent nature of its regulation and effects [18, 29, 32].

Therapeutically, both direct inhibition of heparanase and modulation of its downstream pathways represent promising strategies. Non-anticoagulant heparin derivatives, small-molecule inhibitors, and gene-silencing technologies have demonstrated efficacy in preclinical studies. However, clinical trials are still lacking, and the long-term safety and efficacy of heparanase-targeted interventions in cardiovascular disease remain to be established [67].

Future research should focus on several key directions. Longitudinal clinical studies are needed to clarify how circulating heparanase levels evolve across different stages of cardiovascular disease and in response to therapy. Genetic and epigenetic studies may help identify polymorphisms or regulatory mechanisms that influence heparanase expression and enzymatic activity, providing insights into interindividual variability. Furthermore, integrating heparanase into multi-biomarker panels alongside markers of inflammation and endothelial dysfunction could enhance cardiovascular risk stratification and guide precision therapy. Collectively, these efforts will advance the translational potential of heparanase-targeted strategies in cardiovascular medicine.

## Conclusion

Heparanase plays a pivotal role in cardiovascular pathophysiology, functioning as a key modulator of endothelial integrity, extracellular matrix remodeling, and inflammatory activation. Its dysregulation contributes to plaque instability, thrombosis, myocardial fibrosis, and vascular remodeling across multiple cardiovascular conditions. At the same time, its physiological expression remains essential for tissue repair and homeostasis, defining a fine balance between protection and injury.

Heparanase represents both a challenge and an opportunity in cardiovascular medicine. Its dual biological natüre (protective at physiological levels through maintenance of tissue remodeling and repair, yet pathogenic when overexpressed, driving inflammation, fibrosis, and thrombosis) complicates therapeutic targeting. Moreover, the absence of highly selective and tissue-specific inhibitors limits clinical translation, as systemic suppression risks interfering with normal endothelial turnover and wound healing. These factors underscore the need for precise modulation strategies rather than broad inhibition.

Overall, understanding heparanase as both a pathogenic mediator and a physiological regulator broadens its relevance beyond molecular biology, positioning it as a potential biomarker and therapeutic target. Translating this growing knowledge into clinical benefit depends on developing selective modulators that preserve vascular homeostasis while counteracting the enzyme’s detrimental effects in disease.

## Data Availability

No datasets were generated or analysed during the current study.
